# Application of Fungus Enzymes in Spent Mushroom Composts from Edible Mushroom Cultivation for Phthalate Removal

**DOI:** 10.3390/microorganisms9091989

**Published:** 2021-09-19

**Authors:** Bea-Ven Chang, Chiao-Po Yang, Chu-Wen Yang

**Affiliations:** Department of Microbiology, Soochow University, Taipei 11102, Taiwan; bvchang@scu.edu.tw (B.-V.C.); paul841003@gmail.com (C.-P.Y.)

**Keywords:** fungus enzymes, mycoremediation, phthalates

## Abstract

Spent mushroom composts (SMCs) are waste products of mushroom cultivation. The handling of large amounts of SMCs has become an important environmental issue. Phthalates are plasticizers which are widely distributed in the environment and urban wastewater, and cannot be effectively removed by conventional wastewater treatment methods. In this study, SMCs are tested for their ability to remove phthalates, including benzyl butyl phthalate (BBP), di-n-butyl phthalate (DBP), and diethyl phthalate (DEP). Batch experiments reveal that BBP, DBP, and DEP can be degraded by the SMC enzyme extracts of four edible mushrooms: *Pleurotus eryngii*, *Pleurotus djamor*, *Pleurotus ostreatus*, and *Auricularia polytricha*. Potential fungus enzymes associated with BBP, DBP, and DEP degradation in SMCs (i.e., esterases, oxygenases, and oxidases/dehydrogenases) are uncovered by metaproteomic analysis using mass spectrometry. Bioreactor experiments indicate that the direct application of SMCs can remove BBP, DBP, and DEP from wastewater, through adsorption and biodegradation. The results of this study extend the application of white-rot fungi without laccases (e.g., *Auricularia* sp.) for the removal of organic pollutants which are not degraded by laccases. The application of SMCs for phthalate removal can be developed into a mycoremediation-based green and sustainable technology.

## 1. Introduction

At present, edible mushrooms are typically grown using a mixture of wood shavings, soybean meal, corn meal, and rice bran in plastic bags. At least 5 kg of spent mushroom compost (SMC) is produced after the production of 1 kg of mushrooms [1]. Therefore, the handling of large amounts of SMCs has become an important environmental issue.

The application of fungi and their derivatives for environmental pollutant bioremediation (called mycoremediation) is a low-cost, effective, and eco-friendly strategy [2]. White-rot fungi have an extracellular enzyme system, which includes ligninolytic enzymes (e.g., laccase, manganese peroxidase, lignin peroxidase, and versatile peroxidase), cellulolytic enzymes (e.g., cellulases and hemicellulases), esterases, oxidases/dehydrogenases, and oxygenases [3]. The laccase-redox-mediator system is one of the most-investigated enzyme reactions in ligninolytic enzyme systems [4,5]. This system has been used to remove many emerging contaminants that are difficult to decompose, such as dye-based industrial pollutants and endocrine-disrupting compounds [6,7,8,9]. Moreover, among the lignocellulytic enzymes, cellulases have been found to have significant potential for industrial applications, especially in the food, chemical, detergent, cosmetics, and pulp and paper sectors, among others [10]. Many edible mushrooms are white-rot fungi. Spent mushroom composts (SMCs) of edible mushrooms, which containing residual mycelia that can continuously produce extracellular enzymes, can be used to degrade organic pollutants such as acetaminophen, sulfonamides, and tetrabromobisphenol-A [11,12,13,14]. However, little is known about the potential of other enzymes (e.g., esterases, oxidases/dehydrogenases, and oxygenases) for the biodegradation of organic pollutants.

Phthalates are esters of phthalic acid and aliphatic alcohol, which are used to improve the softness, flexibility, and extensibility of plastic products. Phthalates, such as benzyl butyl phthalate (BBP), di-n-butyl phthalate (DBP), dicyclohexyl phthalate (DCP), diethyl phthalate (DEP), and di-(2-ethylhexyl) phthalate (DEHP), which are mainly used as plasticizers, have been mass-produced and are widely used in daily life, including in furniture, plastic products, cosmetics, and food packaging [15,16,17,18,19]. Due to their physical and chemical properties, phthalates are easily released into the environment from plastic products [20]. They are widely distributed and have been detected in various environmental compartments, such as sediments, water bodies, soils, and aquatic organisms [21,22,23,24,25]. For humans, phthalates of higher molecular weights may be ingested, while phthalates of lower molecular weights may enter by inhalatory and dermal pathways. Continuous exposure to phthalates may lead to the inhibition of liver-detoxifying enzymes and liver dysfunction [26]. Several in vitro and in vivo studies have indicated that phthalates can cause reproductive and developmental toxicities, by acting as endocrine disruptors [27]. Moreover, phthalates can pass through the placental barrier, thus affecting the developing fetus [28]. DBP has been shown to induce androgen-independent adverse outcomes and anti-androgenic effects among different mammalian models [29]. Studies have revealed that, in the presence of allergens, phthalates can act as adjuvants to induce respiratory and inflammatory responses in rodents [30]. Therefore, the development of methods for environmental phthalate removal is important.

The biodegradation of phthalates has been investigated in several studies [31,32,33,34]. The main basis of phthalate biodegradation pathways is composed of two stages: The first stage is initiated with ester hydrolysis reactions, which generate phthalic acid (PA) and alcohols. Esterases are the key enzymes in this initial stage. The biodegradation is continued with benzene ring cleavage of PA and metabolites produced from the decomposition of PA and alcohol. Several enzymes, including decarboxylases, oxygenases, and oxidases/dehydrogenases may be involved in the second stage [31,32,33,34]. The genus *Pleurotus* comprises a group of edible mushrooms with important biotechnological and environmental applications [35]. The cultivation of *Pleurotus* sp. is economically important in the food industry worldwide [36]. Several studies have shown that phthalates (e.g., dimethyl phthalate, DMP; DEP; and BBP) can be degraded by *Pleurotus ostreatus* [37,38,39]. The pathways of phthalate biodegradation by *Pleurotus ostreatus* have been identified [40,41]. The basis of the phthalate biodegradation pathway by *Pleurotus ostreatus* is similar to that of previously identified phthalate biodegradation pathways. The application of SMCs for phthalate removal can, thus, be developed into a mycoremediation-based green and sustainable technology.

The aim of this study was to evaluate the removal efficiency of phthalates (BBP, DBP, DCP, DEP, and DEHP) using the SMCs of edible mushrooms (*Pleurotus eryngii*, *Pleurotus djamor*, *Pleurotus* *ostreatus*, and *Auricularia polytricha*). Batch experiments were performed in order to identify useful SMCs for phthalate removal. Fungus enzymes in SMCs were analyzed by mass spectrometry to identify enzymes associated with phthalate degradation, and bioreactor experiments were conducted to simulate the direct use of SMCs for the removal of phthalates in reverse osmosis (RO) water and wastewater (wastewater treatment plant effluent).

## 2. Materials and Methods

### 2.1. Chemicals

**Figure 1:**
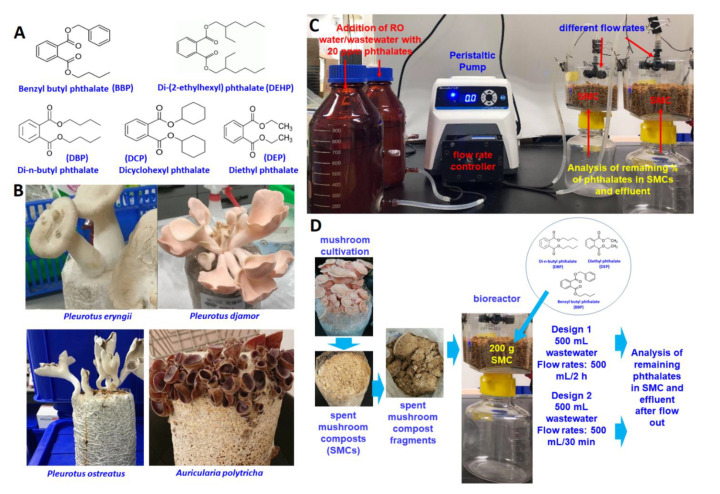
Chemical structural formulas of five phthalates used in this study (**A**); Spent mushroom composts (SMCs) of four mushrooms (**B**); Experimental setting of bioreactor experiments (**C**); 500 mL RO water/wastewater with 20 ppm phthalate were fed into the bioreactors using a peristaltic pump as a flow rate controller (**D**). Two flow rates (500 mL/2 h and 500 mL/30 min) were used in this study. The remaining percentage of each phthalate in SMCs was determined after 500 mL RO water/wastewater flow through SMCs. SMC: spent mushroom compost.

The chemicals CuSO_4_, MnSO_4_, benzyl butyl phthalate (BBP), di-n-butyl phthalate (DBP), dicyclohexyl phthalate (DCP), diethyl phthalate (DEP), di-(2-ethylhexyl) phthalate (DEHP), acetosyringone (AS), syringaldehyde (SA), 2,2’-azino-bis(3-ethylbenzothiazoline-6-sulfonic acid) (ABTS), vanillin (VA), N-hydroxyphthalimide (HPI), (2,2,6,6-tetramethylpiperidin-1-yl) oxyl (TEMPO), and hexane, along with other solvents (analytical grade, 99.0% purity), were purchased from Sigma-Aldrich (Merck/Millipore Sigma, St. Louis, Missouri, USA). The chemical structures of the compounds used in this study are shown in Figure 1A and Supplementary Table S1. Laccases from three fungi (*Aspergillus* sp., *Rhus vernicifera*, and *Trametes versicolor*) were purchased from Sigma-Aldrich (Merck/Millipore Sigma, St. Louis, Missouri, USA).

### 2.2. Sampling and Spent Mushroom Compost

Wastewater samples were collected from effluent (after secondary treatment) of the Dihus domestic sewage treatment plant, Taipei City, Taiwan. SMCs of four mushrooms (*Pleurotus eryngii*, *Pleurotus djamor*, *Pleurotus* *ostreatus*, and *Auricularia polytricha*) were obtained from a mushroom cultivation farm in Nantou, Taiwan (Figure 1B).

### 2.3. Analysis of Remaining Phthalates in Experimental Samples

For liquid samples, 0.5 mL of the sample (reaction mixture) was mixed with 0.5 mL of hexane. For bioreactor experiments, 0.5 g of SMC fragments was mixed with 0.5 mL hexane. The mixture was vortexed for 10 s and centrifuged at 12,000× *g* for 10 min. The organic phase was collected and filtered using 0.22 μm filters and applied to GC analysis. Samples were analyzed by gas chromatography with a flame ionization detector, GC-FID (Hewlett-Packard, Palo Alto, California, CA, USA) and a J&W DB-1701 GC column (Agilent Technologies, Santa Clara, California, CA, USA). The initial column temperature was set at 45 °C; the temperature increased by 10 °C/min to 260 °C and then increased by 2.5 °C/min to 340 °C. Injector and detector temperatures were set at 270 and 290 °C, respectively. Nitrogen was used as both a carrier gas (flow rate 5.0 mL/min) and a makeup gas (flow rate 20.0 mL/min). The recovery percentages for BBP, DBP, DCP, DEP, and DEHP were 97.1, 98.4, 93.3, 96.3, and 94.9%, respectively. The remaining percentages of phthalates were calculated using the formula: [%] = (residue phthalate concentration/initial phthalate concentration) × 100.

### 2.4. Batch Experiments

SMCs containing mushroom mycelium were broken into fragments. Extracellular enzymes were extracted from SMCs by use of 120 g of SMC fragments and 600 mL of sodium acetate buffer (pH 5.0) on a shaker (120 rpm) at room temperature for 3 h. The mixtures were centrifuged (10,000× *g*, 10 min), and the supernatant was collected and filtered to remove debris using 0.22 μm filters. The supernatants (referred to as SMC enzyme extracts) were used for batch experiments. Batch experiments were performed by the addition of 50 mL of SMC enzyme extract and 2 mg/L of each phthalate in 125 mL flask bottles. Reaction mixtures were incubated at 25 °C on a shaker (120 rpm) in the dark. Samples were taken periodically, in order to analyze the residual phthalate concentrations. Each experiment was performed in triplicate.

### 2.5. Metaproteomic Analysis of Fungus Extracellular Enzymes from SMCs by Mass Spectrometry

A total of 100 mL of SMC enzyme extract was concentrated using a 10K MWCO Centrifugal Device (Pall, Houston, TX); furthermore, 24 μL of concentrated samples were applied to SDS-PAGE (12.5%). Protein in-gel digestion was performed using MS-grade Trypsin Gold (Promega, Madison, WI) overnight at 37 °C. Tryptic digests were extracted using 10 μL Milli-Q water, followed by extraction with 20 μL 50% acetonitrile/0.1% trifluoroacetic acid. The extracts were dried in a vacuum concentrator at room temperature, dissolved in 1 μL of 5% acetonitrile/0.5% trifluoroacetic acid, and analyzed using a Thermo Orbitrap Fusion™ Lumos™ Tribrid™ Mass Spectrometer (Thermo Fisher Scientific Inc., Waltham, Massachusetts, USA). The Proteome Discoverer^TM^ Version 2.2 software (Thermo Fisher Scientific Inc., Waltham, Massachusetts, USA) and Uniprot Fungus database (11,680,304 sequences, 2021.02.01.) were used for protein identification.

### 2.6. Adsorption Experiments

Adsorption experiments were performed using 10 g of SMC, 50 mL of sterile water, and each phthalate at different concentrations (2, 10, 20, and 30 mg/L) in a 125 mL flask. After incubating on a shaker (120 rpm) at 25 °C in the dark for 24 h, the mixtures were centrifuged (5870× *g*, 30 min). Then, 0.5 g of SMC fragments from the pellet was collected and mixed with 0.5 mL hexane. The mixture was vortexed for 10 s and centrifuged in 12,000× *g* for 10 min. The organic phase was collected and filtered using 0.22 μm filters. The residual phthalates were analyzed by GC. The adsorption isotherm for the phthalates were fitted by linear regression; that is, *S* = *K_d_C*, where *C* is the phthalate concentration in the aqueous phase, *S* is the phthalate concentration in the solid phase, and *K_d_* is the adsorption constant. Each experiment was performed in triplicate.

### 2.7. Bioreactor Experiments

The set-up of the bioreactor experiments is shown in Figure 1C,D, in which 500 mL plastic filter funnels were used as reactors. The reactors were fed with 500 mL of RO water or wastewater containing 20 mg/L of each phthalate to flow through 200 g of SMCs at room temperature. Two flow rates—500 mL/30 min and 500 mL/2 h—were used, as controlled by a peristaltic pump. The percentages (%) of phthalates remaining in the solid phase of SMCs and effluents of reactors were determined.

## 3. Results

### 3.1. Tests of Phthalate Degradation using Enzyme Extracts of SMCs

**Figure 2:**
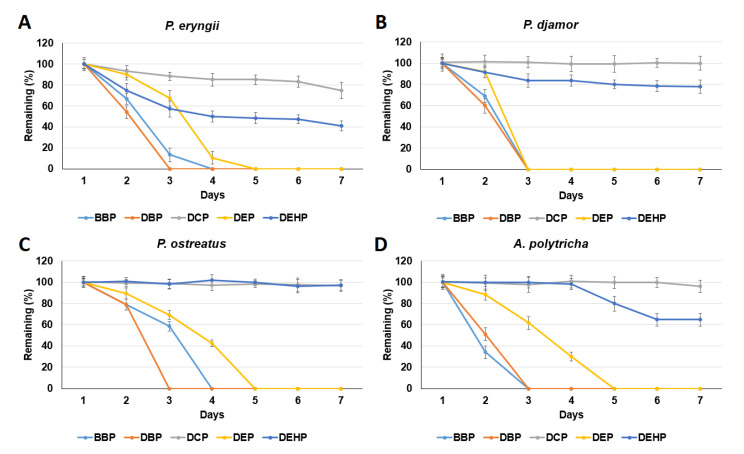
Tests of phthalate (2 ppm) removal using enzyme extracts from spent mushroom compost of four mushrooms: *Pleurotus eryngii* (**A**), *Pleurotus djamor* (**B**), *Pleurotus* *ostreatus* (**C**), and *Auricularia polytricha* (**D**). Data from triplicate experiments are presented as the mean ± SE. BBP: benzyl butyl phthalate, DBP: di-n-butyl phthalate, DCP: dicyclohexyl phthalate, DEP: diethyl phthalate, DEHP: di-(2-ethylhexyl) phthalate.

The phthalate degradation ability of SMC enzyme extracts from the four mushrooms were tested. As shown in Figure 2, the remaining percentages of DCP and DEHP ranged from 74.6–100% and 41.0–97.3%, respectively. The order of the BBP, DBP, and DEP degradation rates for the four SMC enzyme extracts was *P. djamor* > *A. polytricha* > *P. eryngii* > *P. ostreatus*, while the order of the degradation efficiencies was DBP > BBP > DEP. As DCP and DEHP could not be completely degraded, BBP, DBP, and DEP were used in the rest of the experiments in this study.

### 3.2. Tests of Phthalate Degradation using SMC Enzyme Extracts with Mediators

**Figure 3:**
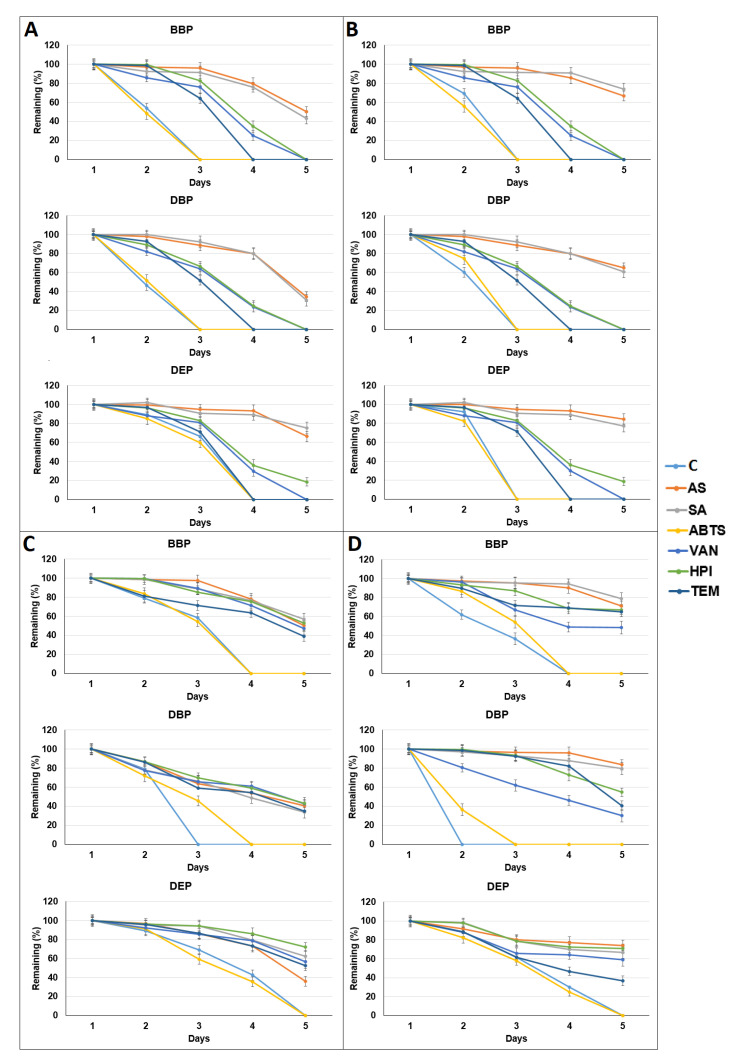
Tests of phthalate (2 ppm) removal using enzyme extracts from spent mushroom compost of four mushrooms: *Pleurotus eryngii* (**A**), *Pleurotus djamor* (**B**), *Pleurotus* *ostreatus* (**C**), and *Auricularia polytricha* (**D**), with the addition of mediators. Data from triplicate experiments are presented as the mean ± SE. C: control, BBP: benzyl butyl phthalate, DBP: di-n-butyl phthalate, DEP: diethyl phthalate, AS: acetosyringone, SA: syringaldehyde, ABTS: 2,2’-azino-bis(3-ethylbenzothiazoline-6-sulphonic acid), VA: vanillin, HPI: N-Hydroxyphthalimide, TEM: (2,2,6,6-Tetramethylpiperidin-1-yl) oxyl.

Six laccase mediators (AS, SA, ABTS, VA, HPI, and TEMPO) were used, in order to test their effects on BBP, DBP, and DEP degradation. As shown in Figure 3, the addition of laccase mediators did not enhance the degradation of BBP, DBP, and DEP in the SMC enzyme extracts. Instead, the degradation of BBP, DBP, and DEP was inhibited with the addition of five of the mediators (AS, SA, VA, HPI, and TEMPO). In some cases, ABTS had no significant effect on the degradation of BBP, DBP, and DEP. The pH values were increased at the end of almost all batch experiments (Supplementary Figure S1). The laccase activities at the end of batch experiments with *P. ostreatus* SMC enzyme extracts were reduced (Supplementary Figure S1B,D,F). In contrast, the laccase activities in the batch experiments with *A. polytricha* SMC enzyme extracts were very low (Supplementary Figure S1H,J,L).

**Figure 4:**
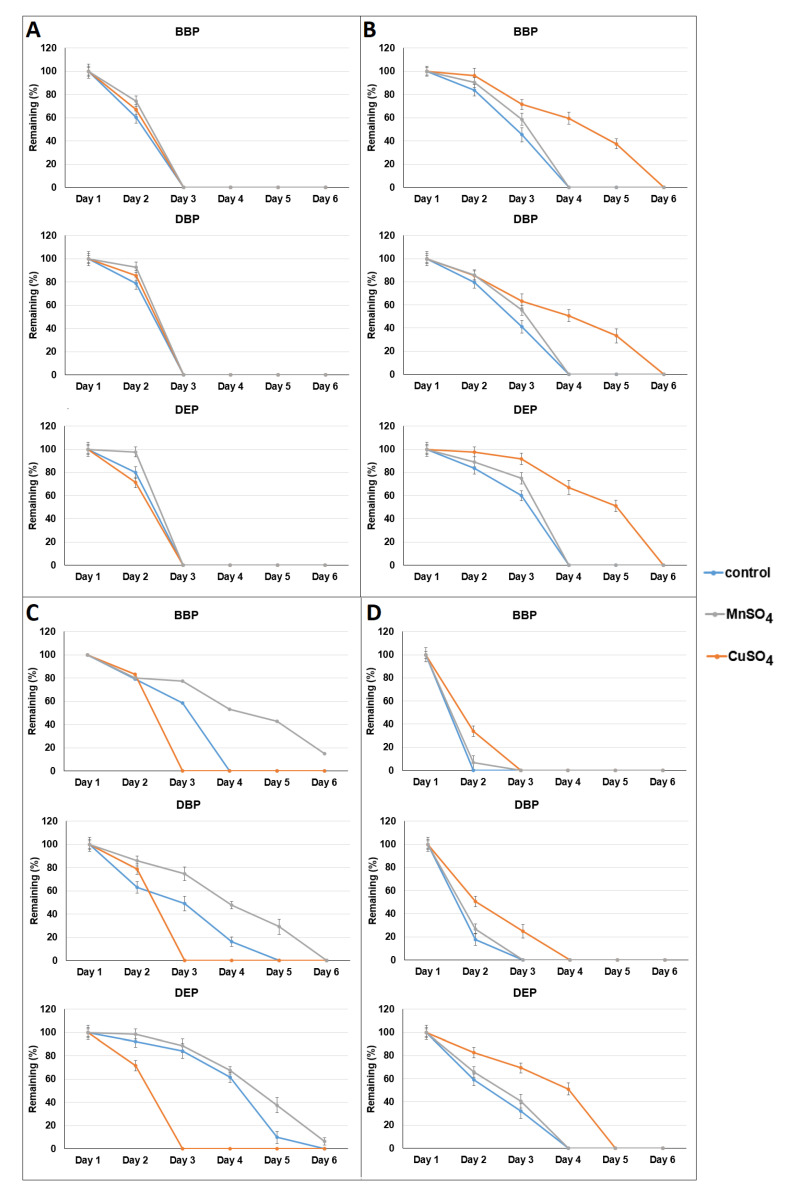
Tests of phthalate (2 ppm) removal using enzyme extracts of spent mushroom compost of four mushrooms: *Pleurotus eryngii* (**A**), *Pleurotus djamor* (**B**), *Pleurotus* *ostreatus* (**C**), and *Auricularia polytricha* (**D**) with the addition of Cu^2+^ (1 mM) and Mn^2+^ (0.5 mM). Data from triplicate experiments are presented as the mean ± SE. Control: without addition of ions, BBP: benzyl butyl phthalate, DBP: di-n-butyl phthalate, DEP: diethyl phthalate.

As shown in Figure 4, the degradation of BBP, DBP, and DEP were enhanced by the addition of Cu^2+^ to the SMC enzyme extract of *P.* *ostreatus*, but were reduced in the SMC enzyme extract of *A. polytricha*. The degradation of BBP, DBP, and DEP were reduced by the addition of Mn^2+^ to the SMC enzyme extract of *P.* *ostreatus*, but it had no significant effect on the SMC enzyme extract of *A. polytricha*. These results suggest that the laccases in SMC enzyme extracts were not involved in BBP, DBP, and DEP degradation. To verify this speculation, laccases from three fungi (*Aspergillus* sp., *Rhus vernicifera*, and *Trametes versicolor*) were used to test the ability of phthalate degradation. As shown in Supplementary Figure S2, BBP, DBP, and DEP were not degraded by the laccases of the three fungi.

### 3.3. Analysis of Fungus Extracellular Enzymes in SMCs

**Figure 5:**
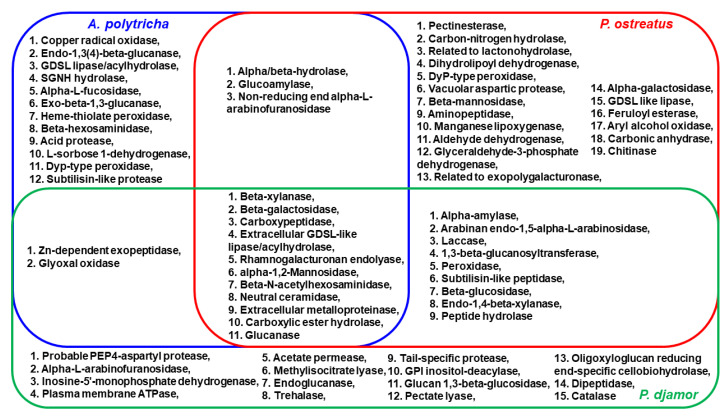
Enzyme composition in the spent mushroom composts of *Pleurotus djamor* (green), *Pleurotus* *ostreatus* (red) and *Auricularia polytricha* (blue).

Fungus extracellular enzymes in SMCs were analyzed by mass spectrometry, in order to identify potential phthalate-degrading enzymes. The complete results are shown in Supplementary Table S2. Overall, 37, 41, and 28 enzymes were identified in the SMCs of *P. djamor*, *P.* *ostreatus*, and *A. polytricha*, respectively (Figure 5). A total of 11 enzymes were present in the SMCs of *P. djamor*, *P.* *ostreatus*, and *A. polytricha*. Laccases were identified in the SMCs of *P. djamor* and *P.* *ostreatus*, but were not identified in the *A. polytricha* SMC. This result was consistent with the results of the batch experiments, as shown in Supplementary Figure S1H,J,L, which indicated a very low level of laccase activity in the *A. polytricha* SMC.

### 3.4. Direct Application of SMCs for Phthalate Removal

**Figure 6:**
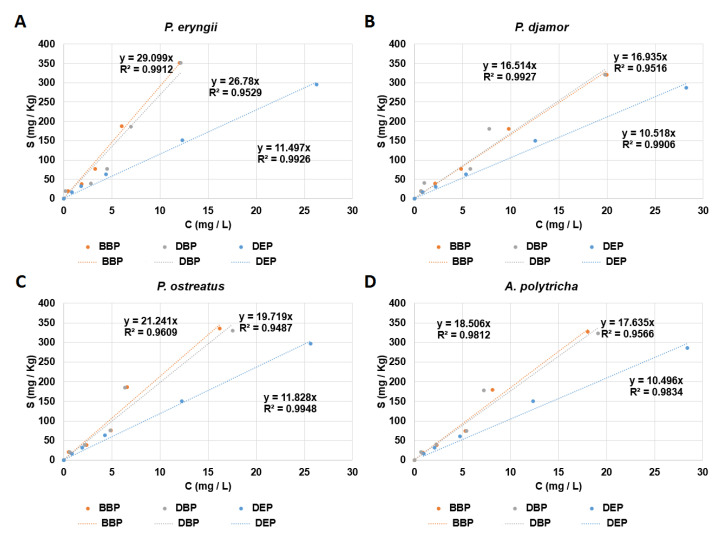
Linear adsorption isotherms of spent mushroom composts of four mushrooms: *Pleurotus eryngii* (**A**), *Pleurotus djamor* (**B**), *Pleurotus* *ostreatus* (**C**), and *Auricularia polytricha* (**D**). The label of *Y*-axis, S, is the concentration of the substrates (BBP: benzyl butyl phthalate, DBP: di-n-butyl phthalate, DEP: diethyl phthalate) in the solid phase. The label of *X*-axis, C, is the concentration of the substrates (BBP, DBP, and DEP) in the aqueous phase.

The production of large amounts of SMC enzyme extracts for wastewater treatment is time-consuming and requires a significant amount of fresh water. Therefore, the direct use of SMCs for phthalate degradation was tested. As shown in Figure 6, the adsorption coefficients of BBP and DBP ranged from 29.1 to 16.5. The range of the adsorption coefficients of DEP was 11.8 to 10.5. These results suggest that the SMCs exhibited a higher adsorption ability for BBP and DBP than for DEP.

**Figure 7:**
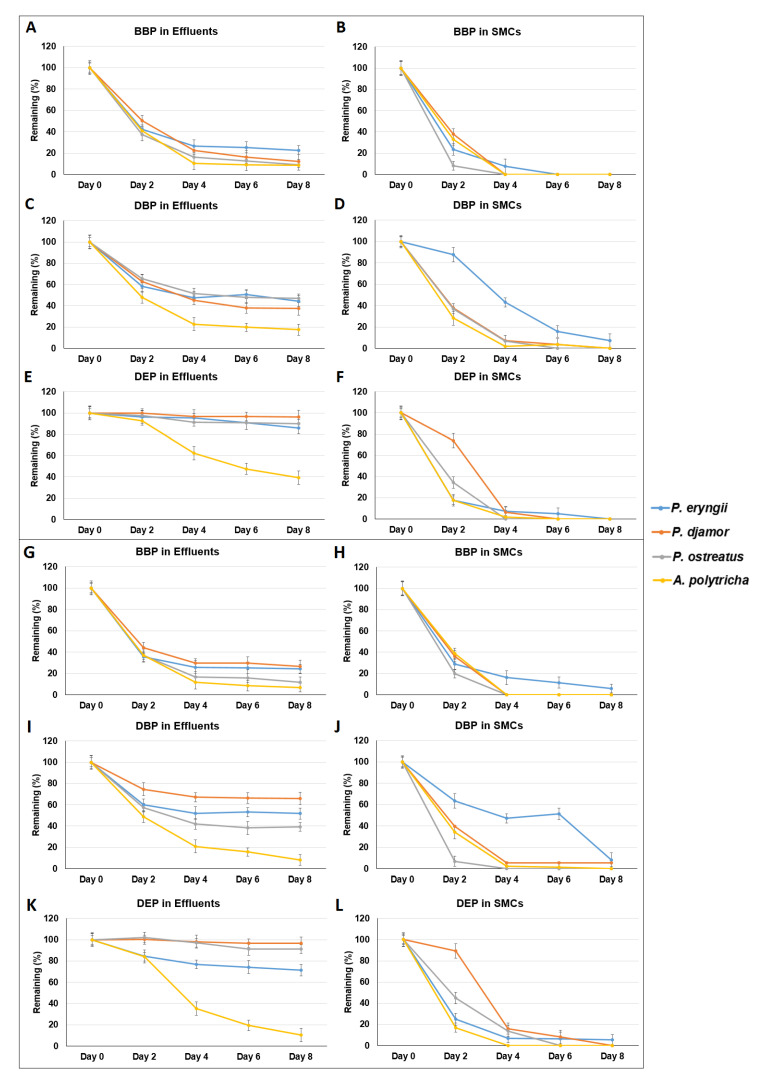
Bioreactor experiments simulating phthalate (20 ppm) removal in RO (reverse osmosis filtration) water (**A**–**F**: 500 mL/30 min, **G**–**L**: 500 mL/2 h) using spent mushroom composts of *Pleurotus eryngii*, *Pleurotus djamor*, *Pleurotus* *ostreatus*, and *Auricularia polytricha*. Data from triplicate experiments are presented as the mean ± SE. S: SMCs, E: bioreactor effluent; BBP: benzyl butyl phthalate, DBP: di-n-butyl phthalate, DEP: diethyl phthalate.

**Figure 8:**
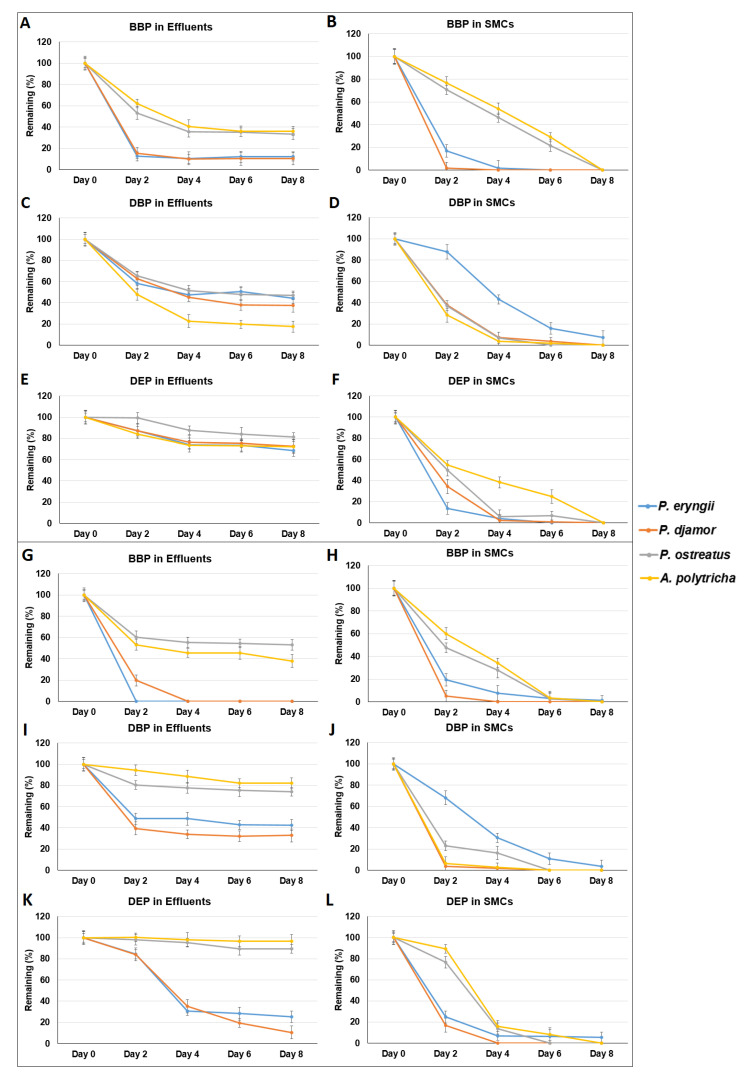
Bioreactor experiments simulating phthalate (20 ppm) removal in wastewater (**A**–**F**: 500 mL/30 min, **G**–**L**: 500 mL/2 h) using spent mushroom composts of *Pleurotus eryngii*, *Pleurotus djamor*, *Pleurotus* *ostreatus*, and *Auricularia polytricha*. Data from triplicate experiments are presented as the mean ± SE. S: SMCs, E: bioreactor effluent, BBP: benzyl butyl phthalate, DBP: di-n-butyl phthalate, DEP: diethyl phthalate.

To simulate the direct removal of phthalates by SMCs, bioreactor experiments were conducted. The remaining percentage of phthalates in SMCs and bioreactor effluents were determined, as shown in Figure 7 and Figure 8. Overall, the remaining percentage of phthalates in SMCs were lower than that in bioreactor effluents. Although wastewater contains many impurities that may have inhibitory effects on the enzymes in SMCs, the removal efficiency for BBP and DBP in wastewater was comparable with the removal efficiency of BBP and DBP in RO water.

## 4. Discussions

Phthalate biodegradation pathways are initiated with ester hydrolysis reactions, which produce phthalic acid (PA) and alcohols. The key enzymes of the initial stage are esterases/hydrolases. The second stage of phthalate biodegradation continues with benzene ring cleavage of PA and the metabolites produced from PA and alcohol decomposition [31,32,33,34]. Dioxygenases and oxidases/dehydrogenases are involved in the second stage of phthalate biodegradation. The results shown in Figure 5 indicate that carboxylic ester hydrolase, pectinesterase, and feruloyl esterase may be the enzymes involved in phthalate ester hydrolysis, while copper radical oxidase, L-sorbose 1-dehydrogenase, dihydrolipoyl dehydrogenase, aldehyde dehydrogenase, and manganese lipoxygenase may be enzymes involved in phthalic acid (PA) and alcohol decomposition. More studies should be performed in order to confirm the roles of these candidate enzymes in phthalate biodegradation.

The results of this study are consistent with previous reports that DEP and BBP can be degraded by *Pleurotus ostreatus* [37,38,39]. The low degradation efficiency of DCP and DEHP (Figure 2) may be due to structural hindrance (by cyclohexyl and 2-ethylhexyl groups, respectively; Figure 1A), preventing esterase/hydrolase binding and reaction.

Mushroom cultivation is economically important in the food industry worldwide. The genus *Pleurotus* comprises a group of edible mushrooms with important biotechnological and environmental applications [35]. The aim of this study is the utilization of SMC (an agro-industrial waste) for the removal of phthalates from wastewater. The results of this study suggest that the extracellular enzymes in the SMCs of *A. polytricha* (although exhibiting very low laccase levels) can be used for BBP, DBP, and DEP removal. Therefore, not only the SMCs of *Pleurotus* sp. (with high levels of laccases), but also the SMCs of *Auricularia* sp. (with very low levels of laccases) can be applied for organic pollutant removal not involving laccases.

The adsorption of phthalates onto SMCs may be affected by two factors: First, the different SMC compositions for different fungi led to different adsorption abilities of the four SMCs with respect to one phthalate (Figure 6). Second, the rates of phthalate-containing water or wastewater flowing through SMCs may cause differences in adsorption. A slow flow rate (500 mL/2 h) may lead to higher phthalate adsorption onto SMCs. These two factors should be considered when applying SMCs for wastewater treatment.

The use of SMCs directly for phthalate removal has three advantages over the use of SMC enzyme extracts: First, the living fungal mycelia in SMCs can continuously produce enzymes to degrade the phthalates adsorbed onto the SMCs. Second, some enzymes may be flushed out with effluents and continuously degrade phthalates in the RO water/wastewater. Third, the preparation of SMC enzyme extracts is time-consuming and requires a large amount of fresh water. In contrast, there are no extra requirements, in terms of water and preparation time, when directly using SMCs.

## 5. Conclusions

SMCs—a mushroom cultivation waste product—were used as low-cost materials for phthalate removal in this study. This design applies the idea of mycoremediation and turns SMCs into a useful resource. Batch experiments showed that BBP, DBP, and DEP can be degraded by the SMC enzyme extracts of *P. djamor*, *P. eryngii, P. ostreatus*, and *A. polytricha*. Enzymes which may be involved in phthalate removal were proposed, based on metaproteomic analysis of fungus extracellular enzymes from the SMCs by mass spectrometry. Bioreactor experiments suggested that the SMCs can be directly used for the removal of phthalates from wastewater. Both adsorption and biodegradation are involved in BBP, DBP, and DEP removal by the direct application of SMCs. The results of this study provide a solution for the removal of phthalates (BBP, DBP, and DEP) from wastewater. Moreover, SMCs with very low levels of laccases (e.g., that from *Auricularia* sp.) can be used for the removal of organic pollutants which are not degraded by laccases.

## Supplementary Materials

The following are available online at https://www.mdpi.com/article/10.3390/microorganisms9091989/s1, Figure S1: Laccase activity and pH values of SMC enzyme extracts from *P. ostreatus* (A–F) and *A. polytricha* (G–L) before and after batch experiments., Figure S2. Tests of phthalate (2 ppm) removal using laccases from *Aspergillus* sp., *Rhus vernicifera*, and *Trametes versicolor*., Table S1: Six mediators used in this study., Table S2: List of extracellular enzymes identified by Mass Spectrometry.
