# Supplementary material for: Application of Fungus Enzymes in Spent Mushroom Composts from Edible Mushroom Cultivation for Phthalate Removal
Source: Microorganisms. 2021 Sep 19;9(9):1989. doi: 10.3390/microorganisms9091989 (PMC8466598; doi:10.3390/microorganisms9091989)
Supplement: Supplementary file 1 [file microorganisms-09-01989-s001.zip › microorganisms-1384447-supplementary/microorganisms-1384447-supplementary-Figure.pdf]

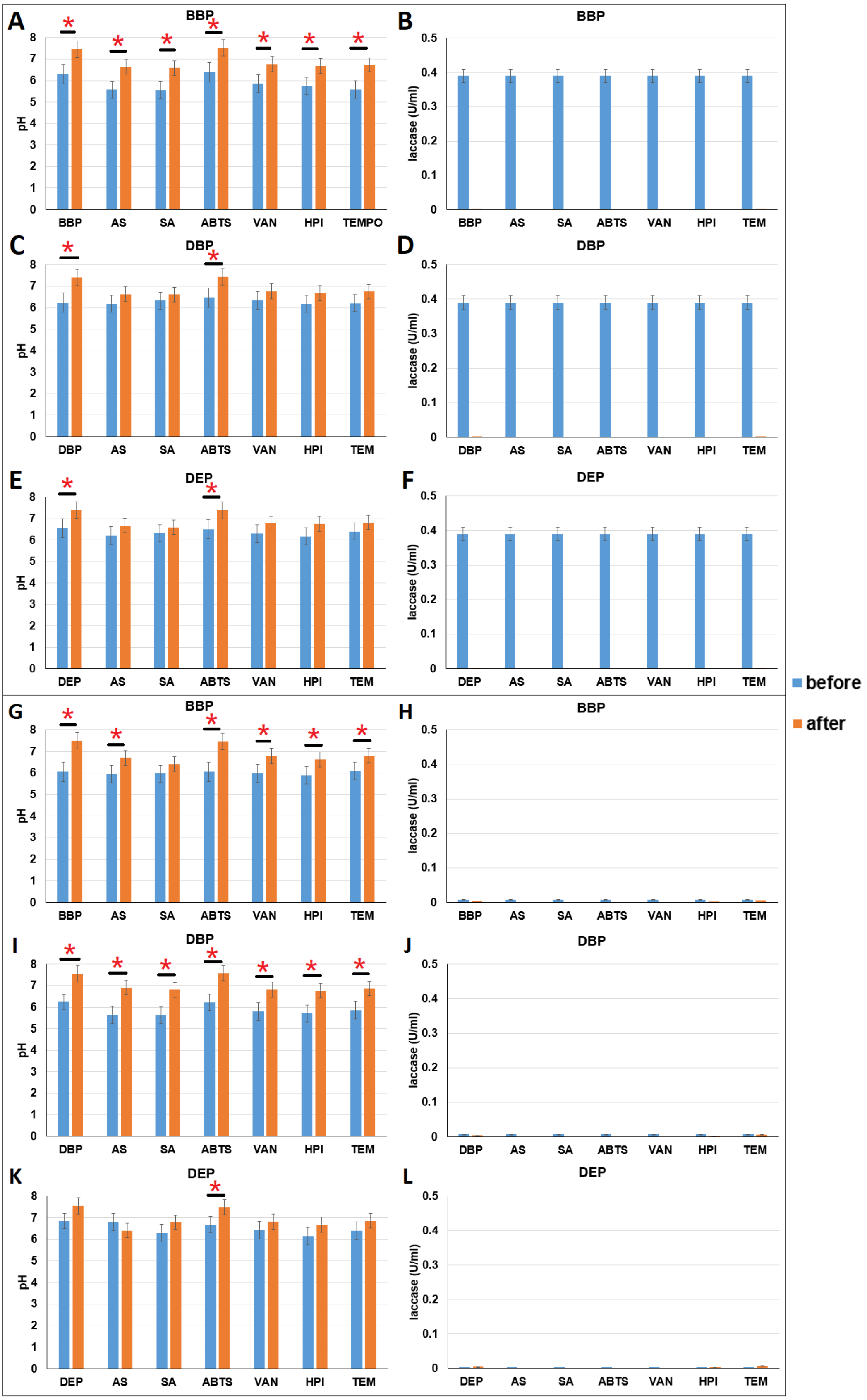

Supplementary Figure S1. Laccase activity and pH values of SMC enzyme extracts from *P. ostreatus* (A–F) and *A. polytricha* (G–L) before and after batch experiments. Red stars indicate the p-values of paired t-tests < 0.05. Data from triplicate experiments are presented as the mean ± SE. BBP: benzyl butyl phthalate, DBP: di-n-butyl phthalate, DEP: diethyl phthalate, AS: acetosyringone, SA: syringaldehyde, ABTS: 2,2'-azino-bis(3-ethylbenzothiazoline-6-sulphonic acid), VA: vanillin, HPI: N-Hydroxyphthalimide, TEM: (2,2,6,6-Tetramethylpiperidin-1-yl) oxyl.

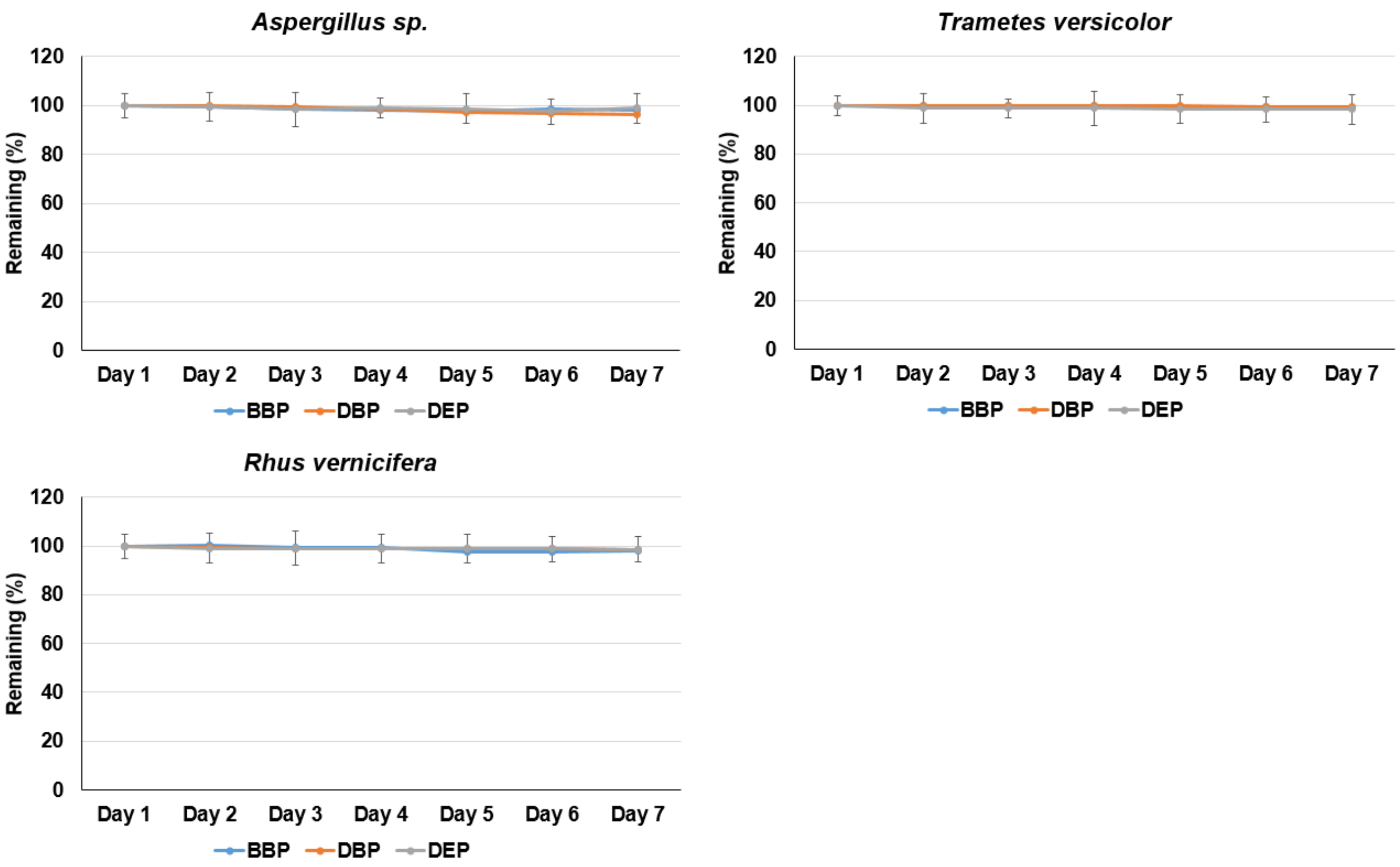

Supplementary Figure S2. Tests of phthalate (2 ppm) removal using laccases from *Aspergillus* sp., *Rhus vernicifera*, and *Trametes versicolor*. BBP: benzyl butyl phthalate, DBP: di-n-butyl phthalate, DEP: diethyl phthalate.
