# Supplementary material for: Application of Fungus Enzymes in Spent Mushroom Composts from Edible Mushroom Cultivation for Phthalate Removal
Source: Microorganisms. 2021 Sep 19;9(9):1989. doi: 10.3390/microorganisms9091989 (PMC8466598; doi:10.3390/microorganisms9091989)
Supplement: Supplementary file 1 [file microorganisms-09-01989-s001.zip › microorganisms-1384447-supplementary/microorganisms-1384447-supplementary-Table.pdf]

Supplementary Table S1. Six mediators used in this study.

Compounds	Abbreviation	CAS No.	Structure
Acetosyringone	AS	2478-38-8	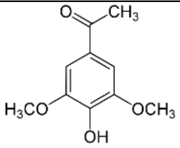
Syringaldehyde	SA	134-96-3	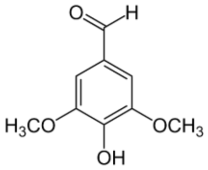
2,2'-azino-bis (3-ethylbenzothiazoline-6-sulphonic acid)	ABTS	30931-67-0	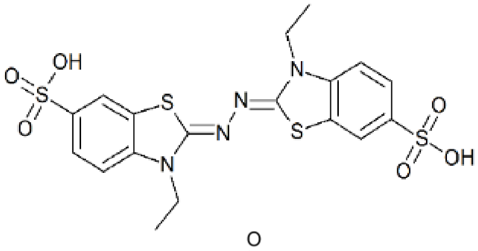
N-Hydroxyphthalimide	HPI	524-38-9	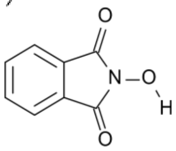
Vanillin	VA	121-33-5	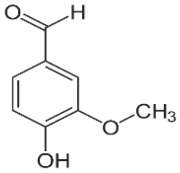
(2,2,6,6-Tetramethylpiperidin-1-yl) oxyl	TEMPO	2564-83-2	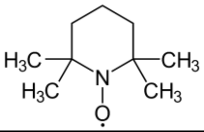
